# Increased chilling tolerance of the invasive species *Carpobrotus edulis* may explain its expansion across new territories

**DOI:** 10.1093/conphys/coz075

**Published:** 2019-11-11

**Authors:** Erola Fenollosa, Sergi Munné-Bosch

**Affiliations:** 1 Department of Evolutionary Biology, Ecology and Environmental Sciences, Faculty of Biology, Universitat de Barcelona, Avinguda Diagonal 643, 08028, Barcelona, Spain; 2 Institute of Research in Biodiversity (IRBio-UB), Universitat de Barcelona, Avinguda Diagonal 643, 08028, Barcelona, Spain

**Keywords:** Aizoaceae, antioxidants, performance, photoprotection, random forest, species distribution model

## Abstract

Invasive plants are expanding their geographical distribution across new regions. Expansion modeling is crucial for geographic prioritization in management policies. However, the assumption of niche conservatism and the lack of information of the species physiological response to the environmental factors determining species presence may hinder predictions. In this study, we aimed to understand the expansion of the widely distributed plant *Carpobrotus edulis* in Europe. We contrasted introduced and native *C. edulis* ecological niches and explored the experimental response to temperature, a major determining factor for species distribution, of native and invasive individuals in terms of different biochemical markers. Niche analysis revealed an expansion of the introduced niche to occupy colder climates. Introduced and native individuals showed differential mechanisms facing low temperatures. Individuals from the native range showed an increased sensitivity to chilling, as reflected by photosynthetic pigment degradation, increased de-epoxidation of xanthophylls and the accumulation of the lipophilic antioxidant alpha-tocopherol. The found physiological differentiation towards an increased invasive chilling tolerance of invasive *C. edulis* individuals together with a high propagule pressure may explain the introduced climatic niche shift to colder climates observed, allowing the extensive expansion of this species in Europe.

## Introduction

Despite invasive species constituting one of the main threats to global biodiversity, the key factors determining their success when colonizing extensive areas are still unknown (Simberloff *et al*., 2013). No single factor explains the species distribution limits and their expansion, but propagule pressure, environmental suitability and biotic relationships may have a role during species expansion across new territories out of their native geographical distribution (Willi and Van, 2019). Besides those factors, the study of invasive species expansion reinforced the importance of rapid evolutionary changes determining the invasive success (Bossdorf *et al*., 2005). In the introduced range, the evolution of functional traits or plasticity might occur as an adaptive response to novel biotic and abiotic environmental pressures. Three light intensities were used to contrast native and invasive populations of *Chromolaena odorata* revealing contrasted phenotypic plasticity between species origin (Liao *et al*., 2019). The comparison of several functional traits of *Acer pseudoplatanus* growing in France (native) and New Zealand (invaded range) under different light regimes revealed also higher plasticity and faster growth of invasive individuals (Shouman *et al*., 2017). Increased biotic tolerance was found to be genetically determined in *Lythrum salicaria* invasive populations, compared to native individuals grown under the same environmental conditions (Joshi and Tielbörger, 2012). Despite genetically contrasted populations between species’ native and introduced ranges, niche conservatism between species’ ranges is one of the assumptions of species distribution modeling (SDM), which constitutes a promising tool to predict suitable areas for the establishment of alien species and the likelihood of the risk of invasion (Peterson, 2003; Thuiller *et al*., 2005; Kearney and Porter, 2009). Moreover, SDM also assumes that actual introduced and native range occurrences define the species’ response to environmental variables (Peterson, 2003), with no clue as to the species’ potential to respond to environmental conditions.

Temperature is one of the main environmental variables limiting plant growth and is therefore a major determining factor in the distribution of plants across different environments (Mittler, 2006). In the case of the invasive species *Amaranthus palmeri,* mean annual temperature has been identified to limit its northward range expansion (Briscoe Runquist *et al*., 2019). Plant response to temperature is complex as several mechanisms at different levels interact to give differential responses accordingly with the magnitude of the temperature change. In fact, cold tolerance is thought to be a difficult trait to acquire, as most angiosperms evolved in tropical climates (Preston and Sandve, 2013). Plant responses may be different in response to temperatures from 15 to 0°C (chilling) from temperatures below water melting point (freezing), which may involve ice formation (Levitt, 1980). Plants experience chilling as part of seasonal events in cold temperate zones and tropical highlands. Plant response to chilling involves alterations in water balance, the suppression of the main metabolic pathways, and an increase of various protective molecules (antioxidants, biomolecule stabilizers and osmoregulators) (John *et al*., 2016). Furthermore, stress by cold conditions is described to lead to reactive oxygen species (ROS) overproduction, capable of severely damaging all the biomolecules and even cause cell death (Suzuki and Mittler, 2006). The synthesis of antioxidants and photoprotective compounds may be a part of a complex reaction mechanism to low-temperature stress involving both protection against and avoidance of ROS production (Cansev *et al*., 2012) and can therefore be used to determine plant sensitivity to cold. A higher capacity to survive and reproduce at different temperatures through this physiological and biochemical adaptations may determine the persistence of a species in a specific location, and could therefore be explaining invasive species success and expansion.

*Carpobrotus edulis* (L.) N.E. Br. (Aizoaceae) is a succulent mat-forming clonal plant native to the Cape Region in South Africa. Despite its restricted native distribution, this species has colonized different coastal Mediterranean regions around the world, becoming an aggressive invader (D'Antonio and Mahall, 1991; Vilà *et al*., 2008). *C. edulis* have shown a great expansion around the Mediterranean Sea, being found in almost all countries of the west Europe, where some of them (Spain, Portugal, France, Italy, Gibraltar and Azores) are considering this species a high threat to their biodiversity (Campoy *et al*., 2018). Clonal propagation has been proposed as a key trait in determining this species’ success (Roiloa *et al*., 2010, 2013, 2014a, 2014b), allowing it to sustain growth in hostile habitats making them habitable after successive death cycles (Fenollosa *et al*., 2016). Moreover, allelopathic compounds released by the species inhibit native species germination, which contributes to the competitive abilities of *C. edulis* (Conser and Connor, 2009; Novoa and González, 2014). Although these traits may contribute to the increased vigor of *C. edulis*¸ few studies have been conducted exploring the differentiation between individuals from native and invasive ranges (Roiloa *et al*., 2016), and none has studied the existence of a differential response to temperature between invasive and native individuals which may be determinant in explaining this species’ expansion.

In the present study, we aimed to understand the *C. edulis* expansion in Europe through an ecophysiological approach. We contrasted introduced and native *C. edulis* ecological niche and explored physiological intraspecific variability of the experimental response to temperature of individuals from both ranges. We explored different physiological responses to low temperatures in terms of different cold-sensitive biochemical markers regarding water balance, photosynthetic efficiency and the content and composition of photoprotective molecules between native and invasive individuals of *C. edulis*. We hypothesized that (i) introduced and native *C. edulis* niches may be different, (ii) individuals from the native and invasive ranges respond differently to temperature, (iii) *C. edulis* has physiological mechanisms to withstand chilling and (iv) species expansion has occurred thanks to a differential physiological response of the introduced and native ranges. First, we analysed the climatic niche of the species to describe the niche dynamics resulting from the invasion process. Secondly, we monitored the response of *C. edulis* to temperature in both an experimental garden and controlled conditions, using individuals from the native (South Africa) and the invasive (Spain) ranges. The combination of the species’ response to a main distribution variable with niche modeling helps us understanding the expansion of *C. edulis*.

## Material and methods

### Climatic niche analysis

To describe the species’ climatic niche and evaluate the existence of differential climatic niches between introduced and native ranges (hypothesis 1), we conducted environmental niche modeling based on the species’ occurrences in its native and European introduced ranges. We used the bioclimatic variables from the WorldClim 2 database at 0.5 arcmin resolution (~1 km^2^) (Fick and Hijmans, 2017), and available presence-only data for *C. edulis* in the Global Biodiversity Information Facility (GBIF) database (http://www.gbif.org, accessed in March 2018 using the ‘dismo’ R package). Occurrences were individually inspected to ensure credibility and geospatial accuracy. Moreover, we removed duplicate occurrences (within 1 km of each other). After filtering, 544 records remained, 492 from Europe and 52 from the native range in South Africa. Climatic niche overlap was quantified on the multivariate environmental space derived from a principal component analysis (PCA) using the WorldClim 2 bioclimatic variables. The entire environmental space of the two studied areas was used to calibrate the PCA and resulted to the PCA-env that was used for the analysis (Broennimann *et al*., 2012). A kernel density function was applied to smooth the occurrences density obtained from the PCA-env, minimizing sampling bias (Warren *et al*., 2008). The Schoener’s D metric was used to estimate niche overlap contrasting the two ranges occupancy of the same climatic space ranging from 0 (no overlap at all) to 1 (complete overlapping) (Schoener, 1970). To test differences between range’s climatic niches, two different tests were used using the niche overlap values: niche equivalence and similarity. Niche equivalence explores the consistence of the niche overlap when randomly reallocating occurrences from both ranges maintaining constant the observed frequencies. Niche similarity addresses whether the environmental niche occupied in one range is more similar to the one occupied in the other range than would be expected by chance. After 1000 iterations, a histogram of simulated values is obtained in both cases and niche equivalency or similarity is rejected if the observed D falls within the 5% density of simulated values.

In order to describe the potential distribution (i.e. the predicted area where the species could be found based on the projection of the climatic niche over the territory) of *C. edulis*, a SDM was build using species occurrences and WorldClim 2 bioclimatic variables. An only-presence maximum entropy model (MaxEnt) was used (Phillips *et al*., 2006, 2018), with 5*k*-fold cross validation. To link the experimental results with the model, we contrast the native SDM (using the occurrences from the native range) with the European SDM (using the occurrences from Europe). Suitability change between SDMs was obtained as raster difference between models. A final mean value of suitability difference in Europe was calculated as:

$$\begin{eqnarray*} & \mathrm{Increased}\ \mathrm{introduced}\ \mathrm{suitability}\nonumber\\ &=\frac{\sum_{\mathrm{i},\mathrm{j}}\left({\delta}_{\mathrm{European}}-{\delta}_{\mathrm{Native}}\right)}{\sum_{\mathrm{i},\mathrm{j}}\left({\delta}_{\mathrm{European}}\right)}\ast 100 \end{eqnarray*}$$

where *δ* stands for model predicted suitability. Increased introduced suitability was calculated considering all *k*-fold combination of native and European SDMs, thus obtaining a variability measure.

### Experimental response to cold temperature of native and invasive individuals

Two experiments to evaluate physiological intraspecific variability species response to winter in the invasive range (Experiment 1) and to evaluate species cold response under controlled conditions (Experiment 2) were performed with native and invasive individuals of *C. edulis* (hypothesis 2 and 3). The realization of both experiments allowed us to confirm the obtained results during natural annual cold (winter) and when temperature is isolated. Seeds from the native and invasive ranges of *C. edulis* were collected in the region of Fish Hoek in South Africa (34°07′S, 18°25′E) and in a protected area in the Cap de Creus Natural Park in Spain (42°21′N, 3°11′E), respectively. About 10 dehydrated fruits from 10 non-connected *C. edulis* clumps at least 10 m apart were collected and stored at 4°C until germination. After seed disinfection with commercial bleach, pooled seeds were germinated under a light cycle of 12:12 h (light:dark hours), at 21°C and 65% humidity. After 4 months, *C. edulis* individuals were transferred into 1 L pots in the greenhouse and morphologically similar individuals from both ranges were placed in the experimental garden of the Faculty of Biology (Barcelona, NE, Spain) under Mediterranean winter conditions of the introduced range to perform Experiment 1. The other individuals were kept in the greenhouse until the chambers were ready for the experiment under controlled conditions (Experiment 2), which was performed in May 2017.

Experiment 1 was designed to monitor the response of *C. edulis* to the low temperatures in its introduced range. At the experimental garden, six individuals per range were sampled at five different points in time from December 2016 (first sampling on 29 December) to February 2017. During January, a cold spell was forecasted and the plants were sampled just before (second sampling point – 13 January) during (third sampling point – 17 January), just after it (fourth sampling point – 24 January) and 1 month after (fifth sampling point – 27 February). During the cold spell, the minimum temperature registered was 1°C. All selected days were clear days and sampling was performed at solar midday to guarantee comparable photoprotective responses. The meteorological conditions registered by the nearest automatic meteorological station (located at 1.4 km) are described in Table 1. Plants were watered two to three times per week according to evapotranspiration demand to ensure water content to reach field capacity. Experiment 2 evaluated the response to low temperatures under controlled conditions, using two identically controlled chambers (Ibercex model E-1350-DV, Madrid, Spain) (light cycle of 12:12 h light:dark). About 20 morphologically similar individuals of *C. edulis* from each range were transferred to the chambers after 3 months of light acclimation. One chamber temperature was kept at ~21°C, whereas the other was set at 8.6°C after 7 days at 14°C (Table 1). Field capacity was ensured by watering plants two times per week. After 7 days at 8.6°C, sampling was performed simultaneously in both chambers at the middle of the photoperiod. The number of replicates was *n* = 5.

**Table 1:** Meteorological conditions for Experiment 1 (experimental garden) and Experiment 2 (controlled conditions). Bold values show samplings with lowest temperatures

	Experiment 1					Experiment 2	
	Dec 29	Jan 13	Jan 17	Jan 24	Feb 27	Control	Cold
Min. temp. (°C)	4.6	**4.7**	**1.0**	**5.8**	9.6	20.5	**7.5**
Mean temp. (°C)	10.3	**10.6**	**4.7**	**8.6**	12.6	21	**8.6**
Max. temp. (°C)	16.4	**13.7**	**7.8**	**14.5**	16.2	23.5	**10**
Max. VPD (mbar)	12.8	**10.4**	**8.4**	**8.5**	6.8	10.7	**4.2**
Max. global solar radiation (W m^−2^)	452	**481**	**497**	**628**	654	181	**181**

Three leaves per plant were taken at midday on sampling days. One was used for assessment of leaf water content and chlorophyll fluorescence measurements, and two were immediately frozen in liquid nitrogen and stored at −80°C until biochemical analysis of photoprotective compounds and antioxidants that describe plant sensitivity to stress by cold conditions. Moreover, stomatal conductance (gs) was measured at solar midday in situ in three young but fully-developed leaves per plant using a leaf porometer (SC-1 Leaf Porometer; Meter Group, Pullman, USA). Light conditions during sampling correspond to the daily maximum global solar radiation described in Table 1 for each sampling day. Leaf water content (H) was calculated as (FW – DW)/DW, where FW is fresh weight and DW dry weight, determined at 60°C at constant weight. To estimate photosynthetic efficiency, chlorophyll fluorescence parameters were determined by the saturation pulse method using a portable pulse amplitude-modulate fluorometer (MINI-PAM photosynthesis yield analyser; Walz, Effeltrich, Germany) on fully developed and illuminated leaves. The relative efficiency of photosystem II (φ_PSII_) was measured under incident irradiation. The maximum quantum yield of photosystem II (*F_v_*/*F_m_*) was measured after a 30-min dark adaptation period. Photosynthetic pigments (chlorophylls *a* and *b*, chlorophyll *a/b* ratio, zeaxanthin, antheraxanthin, violaxanthin, lutein and β-carotene), and α-tocopherol (the main form of vitamin E) were measured as stress markers. The de-epoxidation state (DPS) of the xanthophylls was calculated as (Zx + Ax)/VAZ, where Zx stands for zeaxanthin, Ax for anteraxanthin and VAZ for the sum of violaxanthin, anteraxanthin and zeaxanthin. Pigment content and composition are both good stress markers that reflect plant stress sensitivity and are commonly used in physiological studies (Pintó-Marijuan and Munné-Bosch, 2014). Leaf samples were ground in liquid nitrogen using a mix ball and extracted with cold methanol containing 0.01% butylated hydroxyltoluene using ultrasonication. After centrifuging at 12 000 rpm for 10 min at 4°C, the supernatant was collected and the pellet re-extracted with the same solvent until it was colourless; then, supernatants were pooled and filtered with 0.22 μm and transferred to high-performance liquid chromatography (HPLC) vials. Photosynthetic pigments were separated on a binary-solvent gradient using a reverse-phase HPLC system and quantified with a diode array detector, as described by Munné-Bosch and Alegre (2000). Alpha tocopherol was separated isocratically in a normal-phase HPLC system and quantified with a fluorescent detector, as described by Amaral *et al*. (2005). Quantification was based on the results obtained from the fluorescence signal and compared to that of a calibration curve made with an authentic standard (Sigma-Aldrich, Steinheim, Germany).

### Linking physiology and ecological performance

To understand the link between the distribution of *C. edulis* and the species physiological response to temperature and unravel if differential physiological responses determine species expansion (hypothesis 4), Classification and Regression Random Forest analysis were performed to capture non-linear multivariant responses. Random Forest (RF) is a flexible machine learning algorithm that can be used both for regression and classification. Random Forest Classification was performed to capture the physiological differentiation between ranges and Random Forest Regression to predict species distribution. We hypothesized that the relative abundancy of *C. edulis* at different temperatures in Europe may be explained by the observed physiological values in the performed experiments. To contrast that hypothesis, we obtained relative abundancy values from GBIF occurrences (from now on: Performance) linked to a temperature variable. We selected mean temperature of the coldest quarter of the year (BIO11), as it better represents the mean temperature evaluated period in Experiment 1, performed on the coldest quarter of the year. Data from controlled conditions were not used for this analysis, as light conditions were not comparable.

### Statistics

Niche analysis was performed with the *ecospat* package (Broennimann *et al*., 2016). SDM was performed using the packages *dismo* and *rmaxent*. A two-way mixed ANOVA of repeated measures with ‘Range’ as a between-samples factor and ‘Time’ as a within-samples factor was performed for Experiment 1. A two-way ANOVA with ‘Range’ and ‘Treatment’ was performed for Experiment 2. The Tuckey test was used as a *post-hoc* method. Data were tested with Shapiro-Wilk and Levene tests for normality and homocedasticity and transformed whenever necessary. All analyses were performed using the *nlme* and *multcomp* packages in R 3.3.3. Random Forest analysis was performed using *randomForestSRC* and *ggRandomForests* packages, and the workflow suggested in Feld *et al*. (2016).

## Results

### Differential climatic niches between origins

**Figure 1:**
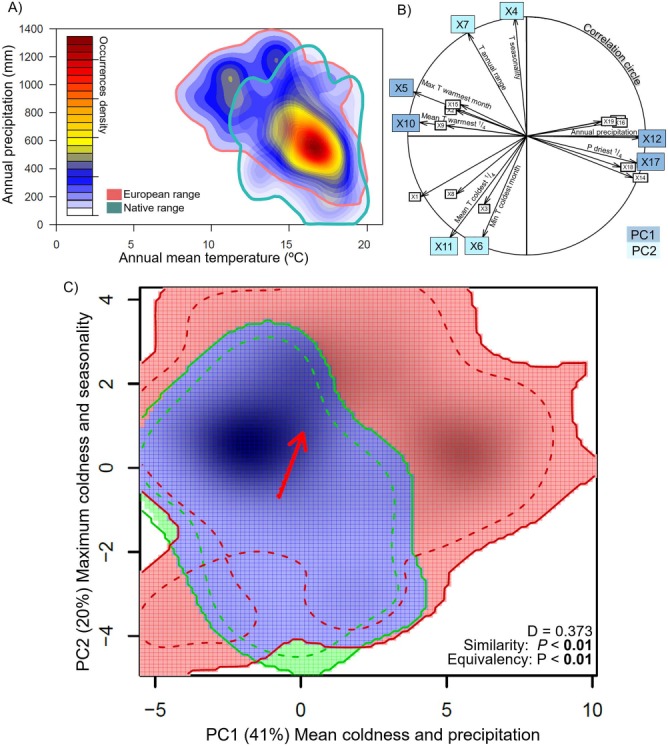
(A) Kernel density estimation for *C. edulis* occurrences in response to annual mean temperature and precipitation. (B) Correlation circle for the PCA-env analysis, with the 19 bioclimatic WorldClim variables (X1-19). Bioclimatic variables full names can be found at: http://worldclim.org/bioclim. (C) Niche dynamics: stability, expansion and unfilling (in blue, red and green respectively) in the multivariate climatic space for native compared to the European niche of *C. edulis* considering the two first components from the PCA-env. D Stands for Schoener’s D overlap value. Solid and dashed lines delineate 100 and 75% of the available background environment, respectively.

Niche analysis revealed a broader introduced niche, expanded to lower temperatures (Fig. 1), supporting our first hypothesis. Considering the European and native ranges, the distribution of *C. edulis* is constrained to annual mean temperatures of between 7 and 20°C and annual precipitation ranging from 100 to 1500 mm (Fig. 1A). PCA-env analysis found two main components, which represent mean coldness and precipitation (PC1) and maximum coldness and seasonality (PC2), as revealed by the weights of the variables (Fig. 1B). The comparison of the niche area plotted onto the multivariate space by the native and the European ranges revealed a broader niche considering the introduced range towards low temperatures (Fig. 1C). Little overlap was found between native and introduced ranges, as Schoener’s *D* = 0.373, meaning that niche overlap between native and introduced ranges is lower than 40%. Niche similarity and equivalence were discarded (*P*-value < 0.05 in both cases) (Fig. 1C), revealing that niches are not equivalent in the different geographical areas and that the introduced niche tends to be more similar to random than native niche.

**Figure 2:**
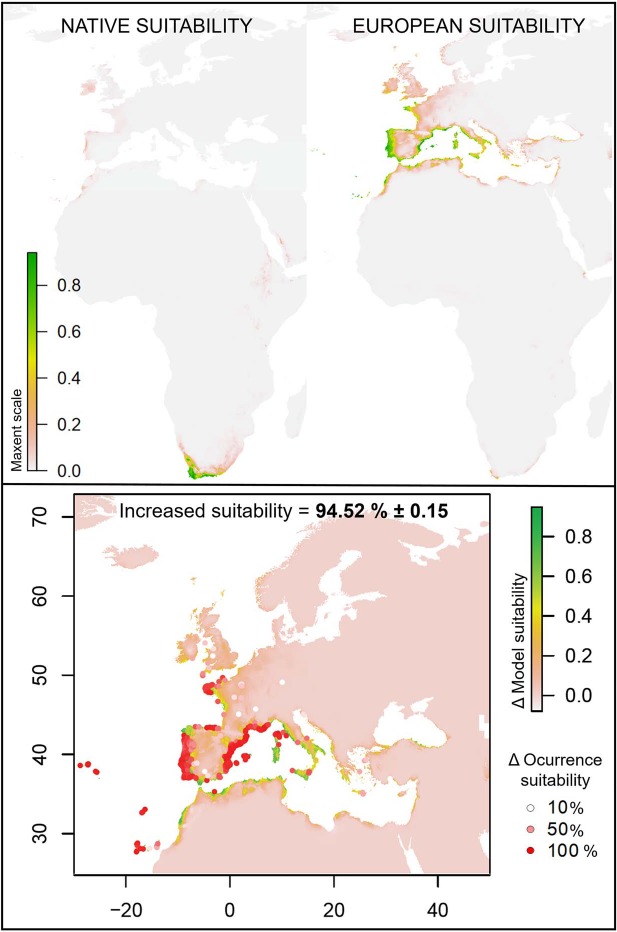
MaxEnt predicted suitability considering the native and the European occurrences of *C. edulis* (Native and European suitability, respectively) and difference between both projections (Increased suitability). Dots correspond to actual invasive occurrences, coloured by the percentage of increased suitability between native and European projections.

The designed SDM for the native and the European *C. edulis* resulted in completely different introduced projections on Europe (Fig. 2). The suitability (0–1) values obtained when projecting the native niche over Europe were extremely low in comparison with the introduced projection. The comparison of both projections revealed a 94.52% ± 0.15 difference at the introduced range. In other words, the niche difference between introduced and native ranges of *C. edulis* leads to extremely different (more than 90%) potentially habitable areas in Europe (Fig. 2).

### Physiological response to chilling

**Figure 3:**
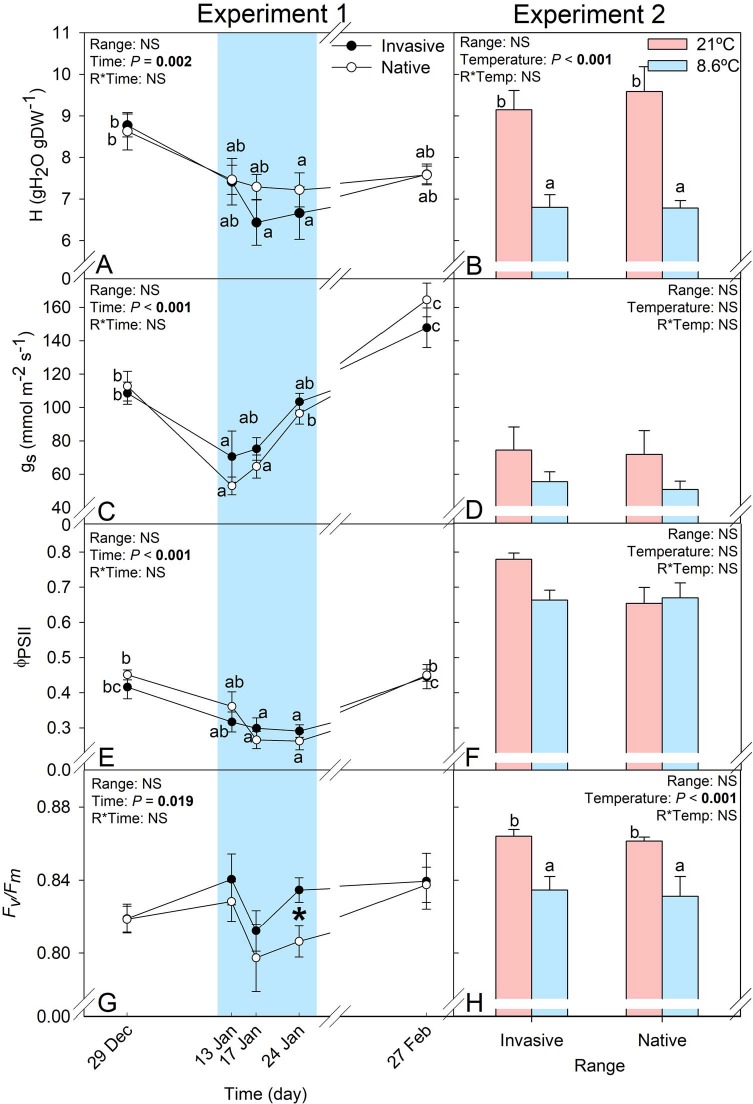
Leaf hydration (H), stomatal conductance (*g_s_*), maximum (*F_v_/F_m_*) and relative (φ_PSII_) efficiency of photosystem II of individuals of *C. edulis* collected in its native (South Africa) and invasive (Spain) ranges in Experiment 1 at experimental garden and 2 under controlled conditions. Blue highlights samplings with lowest temperatures. In the left panels, letters represent significant differences (*P* < 0.05) between sampling dates within each range, whereas in the right panel, represent significant differences between the different bars. Data are shown as mean ± standard error (SE).

Chilling induced alterations in *C. edulis* water balance (Fig. 3A–D). With the arrival of the cold spell, a significant decay was detected in hydration and stomatal conductance. The lowest values of leaf hydration (H) were registered at the lowest air temperatures (7.8°C during sampling). In spite of the fact that hydration remained low during the cold spell, transpiration was restricted only at the beginning. The lowest registered stomatal conductance was 61.73 ± 8.18 mmol m^−2^ s^−1^, which represented a reduction of ~60% from the highest mean value observed (air temperature = 15.6°C). Despite different vapor pressure deficit (VPD) between experiments (Table 1), similar leaf hydration was found in the control individuals under controlled conditions in comparison to the results at the experimental garden, with equivalent reductions when the temperature fell below 10°C (Fig. 3A and B). Despite non-significant differences between treatments, lower values of stomatal conductance were registered for the cold treatments (Fig. 3D). No significant differences (*P* > 0.05) in any of the tested conditions were found between ranges for hydration and stomatal conductance.

**Figure 4:**
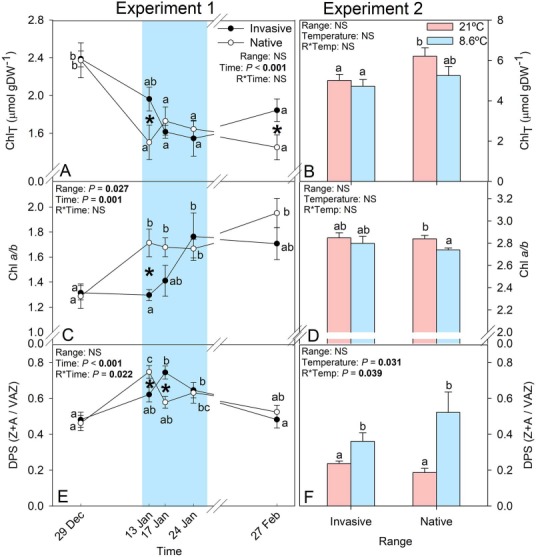
Total chlorophylls (Chl_T_), chlorophyll *a*/*b* ratio and xanthophyll DPS of individuals of *C. edulis* collected in its native (South Africa) and invasive (Spain) ranges in Experiment 1 at experimental garden and 2 under controlled conditions. Blue highlights samplings with lowest temperatures. In the left panels, letters represent significant differences (*P* < 0.05) between sampling dates within each range, whereas in the right panel, represent significant differences between the different bars. Data are shown as mean ± SE.

**Figure 5:**
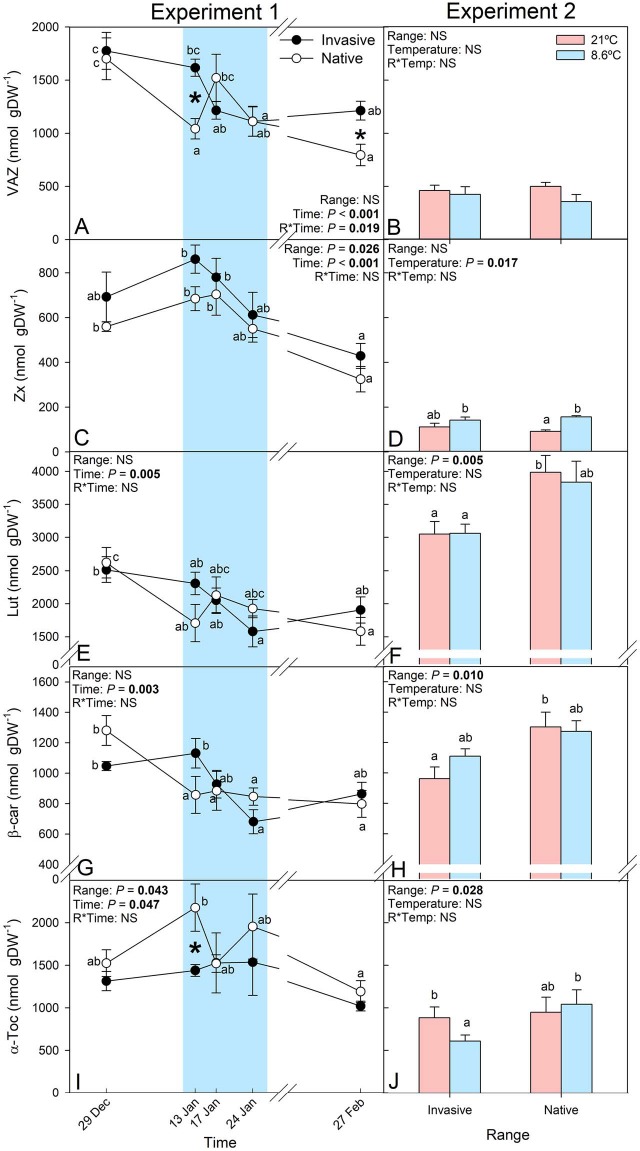
Photoprotective compounds content. Total xanthophyll pool (VAZ), zeaxanthin (Zx), lutein (Lut), β-carotene (β-car) and α-tocopherol (α-Toc) of individuals of *C. edulis* collected in its native (South Africa) and invasive (Spain) ranges in Experiment 1 at experimental garden and 2 under controlled conditions. Blue highlights samplings with lowest temperatures. In the left panels, letters represent significant differences (*P* < 0.05) between sampling dates within each range, whereas in the right panel, represent significant differences between the different bars. Data are shown as mean ± SE.

*C. edulis* individuals from the native and invasive ranges behaved differently in response to chilling regarding photoprotective responses (Figs 3–5). Despite common alterations being observed in both native and invasive *C. edulis*, they occurred at different chilling intensities. Individuals from both ranges showed a significant decrease (~42%) and recovery in the relative efficiency of photosystem II (Φ_PSII_) in response to low temperatures in Experiment 1 (Fig. 3E). No alterations in relative photosystem II efficiency were detected when contrasting both ranges under controlled conditions, under lower irradiation (Table 1). In spite of the fact that no photoinhibition was observed (all registered values of the maximum yield of photosystem II, i.e. *F_v_*/*F_m_*, were above 0.75, Fig. 3G and H), the cold spell induced range differentiation. *C. edulis* from the native range recorded lower *F_v_*/*F_m_* values at the end of the cold spell (Fig. 3G).

Chilling induced changes not only in the photochemical yield but also in the photosynthetic pigments composition and lipophilic antioxidant system (Figs 4 and 5). Both ranges responded to low temperatures with a reduction of chlorophylls, and an increased the proportion of chlorophyll *a* to *b* (i.e. Chl *a/b*), the DPS of the xanthophylls and zeaxanthin content. However, stress indicators such as the chlorophyll content, Chl *a/b*, the xanthophyll pool (VAZ) and its DPS of the two compared ranges of *C. edulis* responded at different chilling intensities leading to significant differences between ranges (Figs 4 and 5A). The DPS increased below 10°C as in Experiment 1 (Fig. 4E and F). The significant zeaxanthin (Zx) content increase in individuals from the native range under cold conditions in Experiment 2 (Fig. 5D) is responsible of the greater DPS increase in the native individuals under cold conditions, in comparison to the invasive individuals (Fig. 4F). Higher contents of lutein (Lut) were found in the native individuals in Experiment 2 (Fig. 5F) despite similar values of this xanthophyll being found in Experiment 1 (Fig. 5E). Despite no significant differences between ranges were found for β-carotene (β-Car) in Experiment 1, mean values at the first sampling point were similar to the values found at 21°C in Experiment 2, where native individuals showed significantly higher β-Car content (Fig. 5G and H). Alpha-tocopherol was found to increase significantly in the native but not in the invasive individuals of *C. edulis* with the arrival of the cold spell (Fig. 5I). Similarly, under controlled conditions, individuals from the native range had a higher α-toc content when temperatures fell below 10°C in comparison to α-Toc content of the invasive individuals (Fig. 5J).

### Differential chilling responses determine species performance

**Figure 6:**
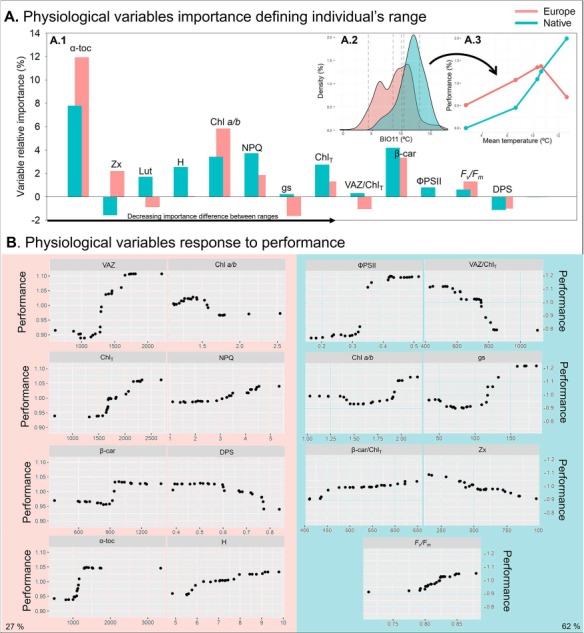
Random Forest classification (A) and regression (B) analysis results for “range” and “performance” prediction respectively. (A.1) Variables relative importance for the European and native models at the classification RF analysis. (A.2) Density of occurrences at different values of mean temperature of the coldest quarter of the year (BIO11) of the European and native ranges. (A.3) Performance at the evaluated experimental temperatures in Experiment 1 of European and native ranges. (B) Important variables selected by the regression RF analysis performed with European and native experimental data. Explained variance in each model is exposed at the bottom extremes of the figure.

The different relative density of occurrences of *C. edulis* under different values of mean temperature of the coldest quarter (BIO11) differed between species ranges, especially under low temperatures between 0 and 5°C, where the native range does not show any occurrence (Fig. 6A.2). The European range showed higher performance in all of the experimental temperatures except for 12.6°C (Fig. 6A.3). On one hand, the obtained Classification Random Forest algorithm revealed differential variable importance for the two ranges (Fig. 6A.1). On the other hand, the obtained Regression Random Forest revealed that 27.3 and 61.7% of the introduced and native performance variability, respectively, was explained by physiological variables (Fig. 6B). The important variables for each model were different, with the exception of Chl *a/b* ratio. Some clear non-linear responses were observed for some variables. VAZ, Chl_T_, β-Car, α-toc and H positively contributed to increased performance in the invasive individuals. For the native individuals, ФPSII, gs, Chl *a/b* and *F_v_/F_m_* positively contributed to performance, whereas Zx and VAZ/Chl_T_ contributed negatively.

## Discussion

Temperature is one of the main environmental variables that define plant species’ distribution, as it constitutes one of the main constrains to plant growth and development, causing stress, damage and even plant death (Mittler, 2006). In our study, the physiology of *C. edulis* was affected by chilling with alterations at multiple levels. The differential chilling sensitivity between ranges exposes the great capacity of this species to adapt to different conditions, allowing this plant to expand its geographical distribution. Photosynthetic efficiency, photosystem composition and antioxidant response showed a consistent pattern of increased sensitivity by the individuals from the native range of *C. edulis*.

The chilling response of *C. edulis* was characterized by water loss, a fall in the relative efficiency of photosystem II, degradation of chloroplast pigments and an increase in the de-epooxidation state of the xanthophyll cycle. Stomatal closure unbalanced redox potential due to photosynthetic substrate limitation and limited photosynthetic efficiency. Individuals of *C. edulis* counterbalanced this alteration at the redox state by modifications in the photoprotective system. The alterations in Chl *a/b* ratio, total chlorophyll content and carotenoid content in *C. edulis* reflect photo-oxidative stress at the thylakoid membrane. It is known that changes in the pigment composition of light-harvesting complexes allow the reduction of absorbed light (with chlorophyll *a* degradation and lower Chl *a/b*) and the increase in energy dissipation through non-photochemical quenching via the xanthophylls, with the deepoxidation of violaxanthin to anteraxanthin and zeaxanthin (the VAZ cycle) preventing excess energy at the chloroplast (Demmig-Adams and Adams, 1996). Degradation of chlorophyll, carotene and xanthophylls has been described as a common response to chilling, revealing cold sensitivity (Kingston-Smith *et al*., 1999). With the exception of Zx, all other carotenoids content decreased in response to the cold spell. Zeaxanthin is known to play a role in thermal dissipation though the VAZ cycle, but an antioxidant role has also been described for this molecule (Havaux *et al*., 2007), which might explain the slight increase in Zx content. The xanthophyll cycle is known to act in some overwintering evergreens during winter as a mechanism to avoid winter photoinhibition (Adams *et al*., 2004). *C. edulis* significantly increased the DPS in response to the cold spell revealing that both ranges use thermal dissipation as quenching mechanisms withstanding low temperatures, supporting our third hypothesis that *C. edulis* has physiological mechanisms to withstand chilling. Previous studies have reported *C. edulis* photoprotective response during winter in the invasive range under natural conditions, concurring with the described increased tolerance to chilling (Fenollosa *et al*., 2017).

The chlorophyll and VAZ decrease and the increase in the chl *a/b* ratio and the DPS were found sooner (at lower chilling intensities) in native *C. edulis* individuals, showing significant differences between ranges at the arrival of the cold spell. Moreover, the transient increase in α-Toc of the native individuals with the arrival of the cold spell, suggests that this molecule might mitigate an oxidative burst derived from the sudden changes in temperature ranges, as this lipophilic antioxidant has an important role scavenging oxygen singlet, preventing lipid peroxidation (Munné-Bosch, 2005). The fact that no alterations in this compound were found under controlled conditions may be a consequence of gradually dropped temperatures, allowing the plants to acclimate to chilling, as chilling sensitivity is described as being dependent on the duration of the stress (Chen *et al*., 2014). The differential response of invasive and native individuals was also supported by both classification and regression random forest analysis, as ecological performance was predicted with different variables for introduced and native models. Regarding our second hypothesis, we conclude that invasive and native individuals of *C. edulis* respond differently to chilling.

Our results of the ecological niche dynamics suggest an introduced niche expansion of *C. edulis* into colder regions, supporting out first hypothesis that introduced and native *C. edulis* have different climatic niches, as similarity and equivalence between niches were significantly discarded. Moreover, almost all introduced areas in Europe are totally unexpected when modeling species distribution considering native occurrences, exposing the importance of this different physiological response. Despite the generally assumed importance of climate match between the native and introduced regions for invasive success (Keane and Crawley, 2002; Wan and Bonser, 2016), we found evidence of differentiated responses to temperature between individuals from native and introduced ranges of *C. edulis* that are consistent with the observed niche shift, supporting our fourth hypothesis that species expansion has occurred thanks to a differential physiological response of the introduced and native ranges. High niche stability might be determinant for the species’ naturalization, but increased cold tolerance may be determinant for this species’ expansion and its aggressive behaviour in its introduced range. In spite of the fact that niche conservatism between native and invasive ranges has been largely assumed and niche shifts in plant species may be rare (Petitpierre *et al*., 2012), recently functional shifts have also been described in invasive species such as *A. pseudoplatanus*, which presented a differential plasticity in response to shade tolerance (Shouman *et al*., 2017). Invasive *Acacia* and *Eucalyptus* trees have also broader physiological niches than native ones (Higgins and Richardson, 2014). Climatic niche shifts in invasive species have also been described for *Centaurea maculosa* (Broennimann *et al*., 2007) and for different exotic species in Australia (Gallagher *et al*., 2010). The different physiological response observed in *C. edulis* toward greater chilling tolerance contributes to discarding the niche conservatism hypothesis, and suggests genetic differentiation between ranges. This is also supported by a recent study contrasting native and invasive individuals of *C. edulis*, which reported that this species’ capacity of division of labor may have been subjected to evolutionary adaptation in the invaded range (Roiloa *et al*., 2016). Rapid evolutionary changes such as genetic drift and inbreeding in founder populations may contribute to explaining invasive niche shifts contributing to local adaptation (Sork, 2017). Many invasive plants appear to grow more vigorously in their introduced than in their native range, and genetic and phenotypic differentiation has been described for some species between ranges (Bossdorf *et al*., 2005). Over the last decade, several studies have demonstrated that rapid adaptation can occur in short time-scales and fuel up the expansion of invasive species into new regions (Sax *et al*., 2007). A recent study with the clonal invasive plant *Alternanthera philoxeroides* in China contrasted individuals from the central portion and the northern edge of the range of this species (where it has expanded recently) and found genetically based differential cold tolerance between individuals (Liu *et al*., 2019). As purposed by the authors, both genetic and epigenetic changes may have a role on this fast genetic differentiation. Epigenetic contribution when facing novel environmental conditions encountered with range expansion may be especially important in clonal species, as they have reduced epigenetic resetting due to the lack of meiosis (Verhoeven and Preite, 2014). Indeed, epigenetic changes together with senescence of plant parts and phenotypic variation have been purposed as parental generation heritance pathways contributing to *C. edulis* success (Fenollosa *et al*., 2016).

Although the climatic niche expansion towards colder zones goes together with the found differential chilling tolerance, the fact that *C. edulis* is able to grow in zones out of its native distribution range may be assisted by a high propagule pressure. Indeed, the lower explained variance of the performance model for the introduced *C. edulis* population (27% in front 62% in the native model) (Fig. 6) suggests that other factors besides cold tolerance are determining the species presence at the introduced range. The study of the invasive marine clonal macrophyte *Caulerpa cylindracea* revealed that propagule pressure together with competence and vegetative growth are determining species invasive success (Balestri *et al*., 2018). The introduction and initial spread of *C. edulis* follows the horticultural industry both also was used for soil and sand dune stabilization (Campoy *et al*., 2018). As evaluated by Bazzichetto *et al*. (2018), this species is now widely planted as an ornamental plant in summerhouses with gardens directly facing coastal dunes providing local input of propagules that constantly supports invasion. Moreover, artificial surfaces had been identified as important propagule sources for *C. edulis* (Carranza *et al*., 2010). The constant human introduction and maintenance of a species out of its native range may promote an increased selection pressure (Willi and Van, 2019) that could have originated the increased chilling tolerance in *C. edulis*. Bottleneck effect and the intrinsic capacity of the species to adapt rapidly to novel environmental conditions though death and growth cycles, clonal growth and epigenetics (Fenollosa *et al*., 2016), could have had also a role on the chilling tolerance acquisition of this species. Moreover, a high hybridization capacity has been described for species of the genus *Carpobrotus* (Albert *et al*., 1997; Suehs *et al*., 2004a, 2004b). In California, hybridization between *C. edulis* and *C. chilensis* contributes to invasion success enhancing species plasticity (Weber and D’Antonio, 2000). In Europe, both *C. edulis* and *Carpobrotus acinacifformis* are considered invasive in some countries and some studies have pointed out the existence of hybridized individuals across the territory, referring to them as *C. affine acinacifformis* (Suehs *et al*., 2004b)*.* There are no studies evaluating the genetic differentiation and genotype prevalence across all the introduced territory of *Carpobrotus* in Europe, but based on species occurrence descriptions, *C. acinaciformis* has a limited distribution in comparison to *C. edulis* (see Fig. 3 in Campoy *et al*., 2018). The found differential physiological response between the analysed native and European *C. edulis* populations in this study goes in accordance with the observed niche shift based on the species occurrence, suggesting that this acquired chilling tolerance may be potentially consistent across the introduced range. However, broad genetic studies are needed to characterize the genetic content of *C. edulis* phenotypes across all its introduced range and understand the role of propagule pressure on the survival of the introduced individuals at the limit of the species distribution.

SDMs have contributed to the projection of invasive species’ spatial dynamics due to climate change, revealing some niche shifts that may require changes in management policies (Romero-Alvarez *et al*., 2017; Wei *et al*., 2017). As climate change will increase the intensity, frequency and duration of abnormally low and high temperatures (Christensen *et al*., 2007), a broad ecological niche may be a key trait to respond to climate change, and therefore more effort should be made to prevent *C. edulis* expansion. However, management strategies may not only consider species growth and expansion but also the effective reduction of propagule pressure, as it may have a determinant role on species expansion and rapid adaptation in the introduced range.

## Conclusions

Our study found evidence of physiological differentiation towards an increased chilling tolerance between individuals from the invaded and native ranges of the species *C. edulis*, which may explain the introduced climatic niche shift to colder climates observed. The species increased chilling tolerance together with a high propagule pressure has allowed its extensive invasion in Europe.
